# Molecular Cloning and Characterization of Carotenoid Pathway Genes and Carotenoid Content in *Ixeris dentata* var. *albiflora*

**DOI:** 10.3390/molecules22091449

**Published:** 2017-08-31

**Authors:** Chinreddy Subramanyam Reddy, Sang-Hoon Lee, Jeong Su Yoon, Jae Kwang Kim, Sang Won Lee, Mok Hur, Sung Cheol Koo, Mi Ran Kim, Woo Moon Lee, Jae Ki Jang, Yoonkang Hur, Sang Un Park, Yeon Bok Kim

**Affiliations:** 1 Department of Herbal Crop Resources, National Institute of Horticultural & Herbal Science, RDA, Eumseong-gun 27709, Korea; suumani@gmail.com (C.S.R.); omega119@naver.com (S.-H.L.); swlee1004@korea.kr (S.W.L.); mok0822@korea.kr (M.H.); ksch992@korea.kr (S.C.K.); milan80623@korea.kr (M.R.K.); wmlee65@korea.kr (W.M.L.); changjk@korea.kr (J.K.J.); 2 Division of Life Sciences, College of Life Sciences and Bioengineering, Incheon National University, Incheon 22012, Korea; 210621036@inu.ac.kr (J.S.Y.); kjkpj@incheon.ac.kr (J.K.K.); 3 Department of Biology, Chungnam National University, 99 Daehak-ro, Yuseong-gu, Daejeon 34134, Korea; ykhur@cnu.ac.kr; 4 Department of Crop Science, Chungnam National University, 99 Daehak-ro, Yuseong-gu, Daejeon 34134, Korea; supark@cnu.ac.kr; 5 Department of Medicinal and Industrial Crops, Korea National College of Agriculture and Fisheries, Jeonju 54874, Korea

**Keywords:** *Ixeris dentata* var. *albiflora*, carotenoid, transcript level

## Abstract

*Ixeris dentata* var. *albiflora* is considered as a potential therapeutic agent against mithridatism, calculous, indigestion, pneumonia, hepatitis, and tumors as well as good seasoned vegetable in Far East countries. Phytoene synthase (PSY), phytoene desaturase (PDS) ξ-carotene desaturase (ZDS), lycopene β-cyclase (LCYB), lycopene ε-cyclase (LCYE), ε-ring carotene hydroxylase (CHXB), and zeaxanthin epoxidase (ZDS) are vital enzymes in the carotenoid biosynthesis pathway. We have examined these seven genes from *I. dentata* that are participated in carotenoid biosynthesis utilizing an Illumina/Solexa HiSeq 2000 platform. In silico analysis of the seven deduced amino acid sequences were revealed its closest homology with other Asteracea plants. Further, we explored transcript levels and carotenoid accumulation in various organs of *I. dentata* using quantitative real time PCR and high-performance liquid chromatography, respectively. The highest transcript levels were noticed in the leaf for all the genes while minimal levels were noticed in the root. The maximal carotenoid accumulation was also detected in the leaf. We proposed that these genes expressions are associated with the accumulation of carotenoids. Our findings may suggest the fundamental clues to unravel the molecular insights of carotenoid biosynthesis in various organs of *I. dentata*.

## 1. Introduction

*Ixeris dentata* var. *albiflora* (Asteracea) is distributed in East Asia—specifically in Korea, Japan, China, and Mongolia. *I. dentata* root and leaf are using as seasoned vegetables in Korea because of its bitter taste. Recently its consumption has been enormously increased due to its root vital therapeutic role in the treatment of mithridatism, calculous, indigestion, pneumonia, hepatitis, and tumors in Korea, China, and Japan [1]. The extracts of *I. dentata* contain crucial secondary metabolites such as cymaroside, aliphatic, triterpenoids, and glycosides [2]. These metabolites play vital role in many physiological activities like, anti-cancer [2], anti-inflammation [3], anti-allergic [4], and anti-hyperlipidemic [5].

Carotenoids are very important secondary metabolites as well as essential health promoting compounds unfortunately, its synthesis does not occur naturally in humans and other animals thus, carotinoids supposed to be acquired through the diet [6]. These are ubiquitously present in nature and represent a group of isoprenoid molecules that has more than 750 pigments [7]. In plants they play crucial roles like lipid membrane stabilization, light (450–470 nm) harvesting in photosynthesis, and photosystem protection from photo-oxidation [8]. Isoprenoid molecules attract the pollinators and seed dispersal agents by exhibiting orange, yellow, and red colors in flowers and fruits [9]. Additionally these molecules are precursors of apocarotenoids that act as signaling compounds, hormones, chromophores, and scent [10]. Among the carotenoids, β-carotene is the precursor of vitamin A, the deficiency of this vital element leads to blindness, xeropthalmia, and premature death [11]. These pigments also have other beneficiary properties like anti-cancer and anti-oxidant [12,13].

**Scheme 1:**
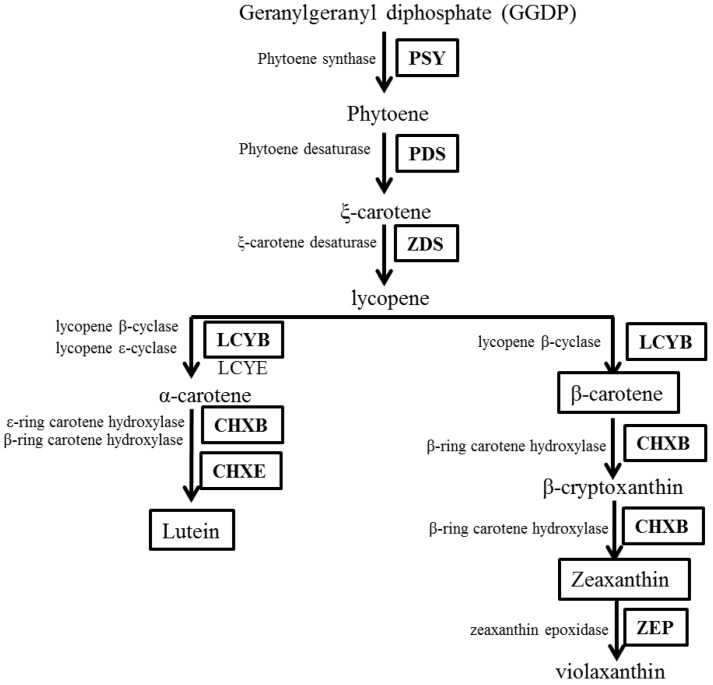
Schematic representation of carotenoid biosynthesis in plants. Carotenoids and their biosynthetic enzymes were analyzed in this study and the analyzed contents were indicated with black boxes. The scheme was generated based on the published literature.

Several medicinal crops EST (Expressed Sequence Tag) repositories have been generated and that have been documented in the EST database at the National Center for Biotechnology Information (NCBI). Numbers of EST libraries were generated for detecting the responsible genes for secondary metabolites biosynthesis and molecular markers development [14]. The NGS tool (Next Generation Sequencing) has been used for genome sequencing, micro RNA expression analysis, and DNA methylation studies due to its robustness and worthwhile technology [15]. Concurrently, functional genes have been detected through de novo RNA transcriptome sequencing, in unfamiliar crops. We generated huge number of transcriptome sequences for *I. dentata* spatial gene expression analysis by RNA-Seq method utilizing an Illumina Hi-Seq 2000 platform. In this study, we identified seven crucial genes that are responsible for carotenoid biosynthesis pathway: phytoene synthase (PSY), phytoene desaturase (PDS), ξ-carotene desaturase (ZDS), lycopene β-cyclase (LCYB), β-ring carotene hydroxylase (CHXB), ε-ring carotene hydroxylase (CHXE), and zeaxanthin epoxidase (ZEP). PSY is the initial and crucial enzyme in this pathway that helps to generate phytoene, from 2 geranylgeranyl diphosphate (GGDP) molecule condensation [16]. PDS helps to convert phytoene in to ξ-carotene and it further turns into lycopene in the presence of ZDS. LCYB is the vital enzyme to convert the lycopene in to β-carotene. Consequently, α-carotene and β-carotene turns into lutein and zeaxanthin respectively by the hydroxylation process, in the presence of CHXB and CHXE. Thereafter, zeaxanthin produces violaxanthin by the help of ZEP (Scheme 1). The comprehensive study of the biosynthesis of carotenoids molecules has elevated our molecular insights into carotenoid biosynthesis mechanisms.

Several studies were elucidated carotenoids accumulation and its related genes transcript regulation in higher plants [8,17,18] and our previous study also revealed it in *Scutellaria baicalensis* [10]. However, carotenoid biosynthesis controlling genes’ spatial expression is not yet reported in *I. dentata*. Thus, in this study, we anticipated elucidation of carotenoid biosynthesis accumulation and its related genes spatial transcriptional regulation in *I. denata* utilizing quantitative real-time PCR (qRT-PCR) and high-performance liquid chromatography (HPLC), respectively.

## 2. Results and Discussion

### 2.1. In Silico Analyses of Carotenoid Biosynthetic Genes from I. dentata

The crucial gene in this pathway *IdPSY* has an open reading frame (ORF) of 1272 bp and that encodes a protein of 424 amino acids. The protein BLAST search analysis was revealed its high sequence similarity to other PSYs (Supplementary Figure S1). IdPSY protein showed maximum identity and similarity with other Asteraceae family members. It shares 87% identity and 100% similarity to *Helianthus annuus* (HaPSY); 82% identity and 98% similarity with *Chrysanthemum boreale* (CbPSY); 92% identity and 85% similarity with *Tagetes erecta* (TePSY); and 84% identity and 98% similarity with *Cynara cardunculus* var*. scolymus* (CcPSY) respectively. It was also shared with other family members like *Camellia sinensis* 78% identity and 99% similarity. The conserved regions of the PSY, trans-isoprenyl diphosphate synthase, for the head-to-head condensation (trans-IPPS-HH) and aspartate rich domains (DXXXD) were also found in IdPSY [10,19,20]. The second gene in this pathway *IdPDS* has an ORF of 1674 bp and it translated a protein of 558 amino acids with 62.28 kDa of a predicted molecular weight (Supplementary Figure S2). IdPDS was 87, 88, 88, 81, and 81% identical to PDS from *Helianthus annuus* (HaPDS), *Chrysanthemum morifolium* (CmPDS), *Tagetes erecta* (TePDS), *Diospyros kaki* (DkPDS), and *Camellia sinensis* (CsPDS) respectively. The signature motifs of the PDS, Carotenoid binding domain and dinucleotide-binding regions (GXGX2GX3AX2LX3GX6EX5GG) were also found in IdPDS (Supplementary Figure S2) [21,22]. Another intermediate gene of this pathway *IdZDS* was comprised an ORF of 1706 bp that encodes a protein of 568 amino acids with 63.171 kDa of predicted molecular mass. IdZDS was shared 96% identity and 95% similarity with *Helianthus annuus* (HaZDS), 94% identity and 96% similarity with *Chrysanthemum morifolium* (CmZDS); 87% identity and 96% similarity with *Daucus carota subsp. sativus* (DcZDS); 87% identity and 97% similarity with *Nicotiana tabacum* (NtZDS); and 87% identity and 97% similarity with *Lycium chinense* (LcZDS). The PDS identical features, N-terminus dinucleotide-binding domain, and C-terminus carotenoid-binding motifs were also found in IdZDS (Supplementary Figure S3) [21,22].

Lycopene β-cyclase is the common enzyme to convert lycopene to α and β carotene, the *IdLCYb* contained 1515 bp length that encoding a protein of 505 amino acids with 56.56 kDa of expected molecular mass. IdLCYb displayed closest homology with *Taraxacum officinale* (ToLCYb), i.e., 96% identity and 100% similarity and followed by *Tagetes erecta* (ToLCYb) 89% identity and 100% similarity; *Vitis vinifera* (VvLCYb) 84% identity and 100% similarity; *Rhododendron kiusianum* x *Rhododendron indicum* (RkLCYb) 83% identity and 100% similarity; and *Chrysanthemum morifolium* (CmLCYb) 87% identity and 100% similarity respectively. The signature motifs of LCYB, β cyclase catalytic activity domains, charged region, di-nucleotide binding site, and cyclase motifs were identified in the IdLCYB (Supplementary Figure S4) [23]. The common gene for both the carotenoid branches, IdCHXB composed an ORF of 1062 bp long and it encodes a protein of 353 amino acids with predicted molecular weight of 39.79 kDa. IdCHXB exhibited close homology with *Vitis vinefera* (VvCHXB) 97% similarity and 71% identity, and *Solanum pennellii* (SpCHXB) *Solanum tuberosum* (StCHXB) 96% similarity and 71% identity; followed by *Nicotiana sylvestris* (NsCHXB) 96% similarity 70% identity, and *Erythranthe guttata* (EgCHXB) 95% and 69% respectively. Spatially conserved, four histidine domains that are believed to participate in iron ion adhesion while hydroxylation takes place are depicted in (Supplementary Figure S5) [24]. The epsilon carotene hydroxylase form *I. dantata* has an ORF 1063 bp long and it encodes for a protein of 353 amino acids with a 33.62 kDa predicted molecular mass. IdCHXE displayed the nearest homology i.e., 100% similarity and 87% identity with *Morus notabilis* (MnCHXE) and *Camellia sinensis* (CsCHXE). Further *Daucus carota* subsp. *sativus*, *Sesamum indicum*, and *Herrania umbratica* exhibited 100% similarity and 85% identity with IdCHXE. The important motifs—center of α helix which can bind to heme (AGHE) and (EGEN) salt bridges—were identified in this sequence (Supplementary Figure S6) [25]. The terminal enzyme zeaxanthin epoxidase from *I. dentata* genes contained a partial length of 1365 bp that translated a protein of 455 amino acids with a predicted molecular weight of 49.88 kDa. IdZEP exhibited the maximum sequence homology with *Chrysanthemum boreale* (CbZEP) at 95% identity and 100% similarity, followed by *Camellia sinensis* (CsZEP) 85% identity and 100% similarity, *Ricinus communis* (RcZEP) 84% identity and 100% similarity, and *Vitis vinifera* (VvZEP) 84% identity and 100% similarity respectively. The signature domains of the ZEP, lipocalin-like proteins and phosphopeptide-binding motifs were also found IdZEP (Supplementary Figure S7) [26,27].

### 2.2. Carotenoid Biosynthetic Genes Spatial Expression Profiles in of I. dentata

**Figure 1:**
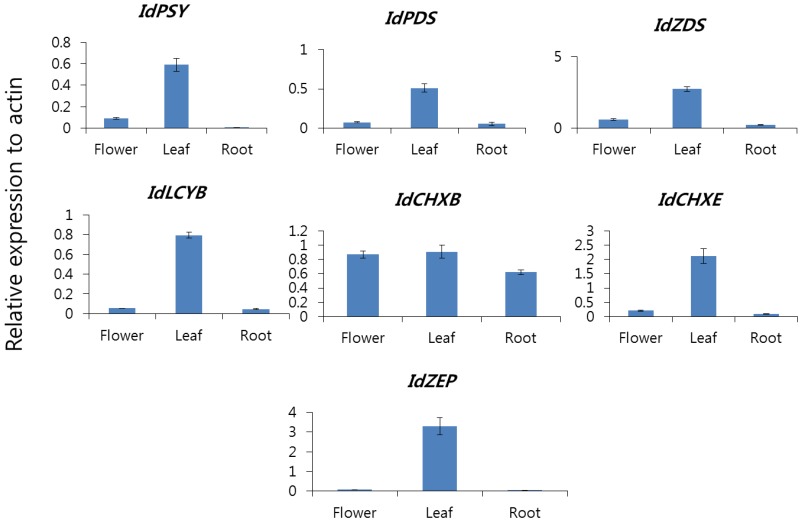
Transcript levels of *IdPSYs*, *IdPDSs IdZDSs*, *IdLCYBs*, *IdCHXBs*, *IdCHXE*, and *IdZEPs* in the flower, leaf, and root of *I. dentata*. The height of the bar and the error bars indicate the mean and standard error, respectively, that was derived from three independent experiments. At least three different samples were used for an individual experiment.

Carotenoid biosynthetic pathway genes spatial expression profiles were investigated in the roots, leaves and flowers of *I. dentata* by qRT-PCR (Figure 1). All the seven genes were highly expressed in the leaf whereas, in the root, minimal or neglected expression profiles were noticed. *IdCHXB* was the only gene expressed constantly in all the organs even here also leaf exhibited maximum expression and followed by flower and root. The terminal gene *IdZEP* showed the highest expression at about 3.5-fold in the leaf followed by *IdZDS* and *IdCHXE* which were expressed 3.0- and 2.5-fold, respectively. Pathway intermediate *IdPDS* and *IdZDS* genes showed similar fashion of expression profiles, i.e., the highest in the leaf and followed by flower and root. The initial and terminal genes, *IdPSY* and *IdZEP*, absolutely have no expression in the root.

### 2.3. Carotenoids Analysis in Different Organs of I. dentata by HPLC

Carotenoids were quantitatively measured and analyzed in different organs of *I. dentata* using HPLC by utilizing the remaining sample as those employed for quantitative real-time PCR experiment (Table 1). Usually massive amounts of carotenoid accumulation takes place in the leaves and the huge quantities of carotenoids were detected in *I. dentata* leaves were β-carotene and lutein. Especially, ample quantity of β-carotene (803.43 μg/g dry weight [DW]) was detected in the leaves, that may elucidates the existence of reasonable quantity of its *cis* isomers, 9 and 13 β-carotenes were (117.03 μg/g DW) and (140.88 μg/g DW). In contrast β-carotene derivative zeaxanthin (21.59 μg/g DW) was found relatively meager in the same leaves. In addition to β-carotene, a great amount of lutein was found in the leaves (415.32 μg/g DW). In the flower, β-carotene and lutein concentrations were vice versa to the leaves. The flower contains significant amount of lutein (197.28 μg/g DW) and a considerable amount of β-carotene (97.26 μg/g DW). The minimal accumulation of β-carotene isomers, 9-*cis*-β-carotene (21.6 μg/g DW) and 13-*cis-*β-carotene (13.41 μg/g DW), were found in flowers. The total carotenoid accumulation in the leaf and flower were (1498.25 μg/g) and (353.59 μg/g DW) respectively, whereas in the root we have not detected any carotenoid.

In this study, we characterized carotenoid biosynthetic pathway genes *PSY*, *PDS*, *ZDS*, *CHXB*, *CHXE*, *LCYB*, and *ZEP*, and also detected carotenoid accumulation in various organs of *I. dentata.* Carotenoid biosynthetic genes were differentially expressed in all organs of *I. dentata.* All the seven genes transcripts were highly expressed in the leaves, whereas in root the transcript level was minimal or not found. The transcript level of *IdCHXB* was high in all the organs. The first enzyme in this pathway PSY is a crucial regulator in carotenoid biosynthesis and accumulation in plants [28,29,30], implying that the *IdPSY*, *IdPDS*, and *IdZDS* minimal expression levels in the root leads to minute accumulation of carotenoids in the roots of *I. dentata*. Abundant carotenoid accumulation was found in the leaf where the expression levels of *IdPSY*, *IdPDS*, and *IdZDS* was maximal. However, lower expressions of *IdPSY*, *IdPDS*, and *IdZDS* were found in the flowers, where a relatively fair quantity of carotenoids were detected. This phenomenon indicates the presence of other PSY isoforms in *I. dentata* which can regulate the flux into the carotenoid biosynthetic pathway in the flowers. Among the two carotenoid branches the β, β-branch contains β-carotene, hydroxylated by CHXB to generate zeaxanthin and further it turns in to violaxanthin in the presence of ZEP. Usually higher expression levels of CHXB and ZEP lead to less accumulation of carotenoids, but our study found conflicting results. The reason might be probably the β, β- branch, *IdCHXB* expression is not sufficient or *IdCHXB* might be an active role in the flux of ε,β-carotenoid branch rather than β, β-branch. Bouvier et al. (1996) showed that the pepper ZEP activity relies on reduced ferredoxin availability [31]. Thus inaccessible, reduced ferrodoxin might be the reason for ZEP limited activity as well as for high zeaxanthin accumulation. Hence, the β-carotene accumulation was also not affected in the leaf of *I. dentata* (803.43 μg/g DW). However, further studies are required to elucidate this phenomena. In general, leaves contain abundant of carotenoids whereas it differs in other organs, thus, the current study also found less quantity of β-carotene in the flower. In another ε, β-carotenoid branch, a huge quantity of lutein was also exhibited in the leaves as well as the flowers of *I. dentata* due to elevated levels of *CHXB* and *CHXE* expression. Carotenoids are richly biosynthesized in green tissues, whereas they are barely biosynthesized in roots of *I. dentata*. This phenomenon is identical to *S. baicalensis* [8], garlic [32], and bittermelon [33] which demonstrates the indispensable role of carotenoids in photosynthetic activity in plants. In addition to roots, abundant source of carotenoids β-carotene (803.43 μg/g DW) and lutein (415.32 μg/g DW) contained *I. dentata* leaf also has been consuming as seasoned food in Far East countries. It has been elucidated that β-carotene has potential roles in the prevention of heart disease, stroke, cancer [34,35,36,37], and disease and it is believed that lutein has a promising role in anti-inflammation and anti-oxidation [38,39].

## 3. Experimental Section

### 3.1. Plant Materials

*I. dentata* var. *albiflora* cultivar was grown in our experimental field in the National Institute of Horticultural and Herbal Science, Rural Development Administration (Eumseong, Korea) in 2015. Different tissue samples (flowers, stems, leaves, and roots) were collected from 15 healthy plants in August 2015. Instantly, all the samples were dipped into liquid nitrogen and further stored at −80 °C until use. The samples were used for RNA extraction and or HPLC ultraviolet analysis. Each replica contains five plants for biological repeats.

### 3.2. Total RNA Isolation and Quantitative Real Time-PCR (qRT-PCR)

Total RNA was isolated from the frozen spatial tissue samples of *I. dentata* utilizing an Easy BLUE Total RNA Kit (iNtRON, Seongnam, Korea). RNA concentration was quantified on a NanoVue Plus spectrophotometer (GE Healthcare Life Sciences, Pittsburgh, PA, USA) and its quality was verified with 1.2% formaldehyde agarose gel electrophoresis by loading 1 μg of total RNA. Consequently, 1 μg of total RNA was used for mRNA conversion using a ReverTra Ace-α Kit (Toyobo, Osaka, Japan) and oligo (dT)_20_ primers. Reverse transcriptase polymerase chain reaction (RT-PCR) procedure was followed as: 11 μL of RNase-free water was mixed with 1 μL of (1 μg) total RNA, 2 μL of 10X buffer, 1 μL of each dNTP (10 mM), 2 μL of oligo dT primer (10 μM), 1 μL of RNase inhibitor (40 u/μL), and 1 μL of reverse transcriptase (4 u/μL) to make final volume of 20 μL. The mixture was kept at 42 °C for 20 min for amplification and RT activity was terminated by heating at 99 °C for 5 min. Furthermore, the cDNA was diluted for 20-fold and it was used for qRT-PCR. Quantitative Real Time-PCR was executed with 20 μL reaction volume that contains 2× Real-Time PCR Smart mix (SolGent, Daejeon, Korea) and 0.5 μM primers. The list of primers used in this study is mentioned in the Table 2. The qRT-PCR program procedure was followed as described earlier [40]. The transcript analysis results were procured using the mean of three samples. The gene of interest was normalized actin gene (GenBank accession No. KY419710). Though we tried with 18S gene, we were unable to get reliable results, thus further continued with actin. Absorbance values were utilized to assess the mRNA copy numbers. The messenger RNA was diluted sequentially for standard mRNA preparation and performed reverse transcription PCR. The mRNA standard values were considered as standard cDNA solutions and the PCR accurate values were analyzed using a free online tool (http://cels.uri.edu/gsc/cndna.html). Different cDNA concentrations were used for qRT-PCR (1 × 10^7^ to 1 × 10^3^ copies for all cDNAs) and the final experiment was conducted thrice with triplicate. Obtained data was analyzed using Bio-Rad PCR machine inbuilt software CFX Manager 2.0 w and the representative values were plotted on log_2_ graph.

### 3.3. Sequence Analysis

*I. dentata* carotenoid biosynthetic pathway genes, deduced amino acid sequences were aligned using Biological Sequence alignment Editor (BioEdit). Protein molecular weight and isoelectric point values were calculated using the compute Mw tool (http://ca.expasy.org/tools/pi_tool.html). Carotenoid biosynthesis pathway gene specific primers were generated through an online program (https://www.genscript.com/ssl-bin/app/primer) (Table 1).

### 3.4. Isolation and HPLC Analysis of I. Dentata Carotenoids

Carotenoids isolation and quantification was performed according to our previous protocol [3,31]. Carotenoids were concisely liberated from the 0.02 g of *I. dentata* sample by the addition of 3 mL ethanol that holds 0.1% ascorbic acid (w/v). Then the mixture was gently vortex for 20 s and kept it, in a water bath for 5 min at 85 °C. Further, the reaction mixture was saponified with 120 μL of potassium hydroxide (80% *w*/*v*) at the same temperature for 10 min. Then, immediately, the samples were placed on ice and added 1.5 mL of deionized cold water. 0.2 mL of β-Apo-8′-carotenal (25 g/mL) was used as internal control in this study. Hexane (1.5 mL) was used for carotenoids extraction and separated the layers by centrifugation at 1200× *g*, the step was repeated once again. Extracted carotenoids were aliquoted and freeze dried in liquid nitrogen, then the sample was redissolved in dichloromethane/methanol (50:50 (*v*/*v*)) before pursuing HPLC analysis. The carotenoid fractionation analysis was carried out in a C30 YMC column (250 × 4.6 mm, 3 μm; Waters Corporation, Milford, MA, USA) by Agilent 1100 HPLC (Massy, Paris, France) that furnished with a photodiode array (PDA) detector. The detection range of carotenoids 450 nm was used, to generate the chromatograms. Solvent A and B were contained, methanol/water (92:8 *v*/*v*) with 10 mM ammonium acetate and methyl *tert*-butyl ether (100%), respectively. Gradient elution analysis (1 mL/min) was executed by the following conditions A 90%, B 10%, 0 min; A 83%, B 17%, 20 min; A 75%, B 25%, 29 min; A 30%, B 70%, 35 min; A 30%, B 70%, 40 min; A 25%, B 75%, 42 min; A 90%, B 10%, 45 min; and A 90%, B 10%, 55 min. CaroteNature’s (Lupsingen, Switzerland) carotenoid standards were used for quantification. Four different concentrations of standard carotenoids were used for raising the calibration curve as per peak ratios of β-apo-8′-carotenal. The obtained linear regression co efficient values for lutein, zeaxanthin, β-cryptoxanthin, 13-*cis*-β-carotene, β-carotene, β-carotene, and 9-*cis*-β-carotene were *y* = 125.46*x* + 83.25, with *r* = 0.984, *y* = 320.23*x* − 18.15, with *r* = 0.999, *y* = 226.22*x* + 23.65, with *r* = 0.996, *y* = 227.27*x* − 24.10, with *r* = 0.999, *y* = 372.42*x* − 53.85, with *r* = 0.999, *y* = 146.29*x* + 124.10, with *r* = 0.957, and *y* = 289.77*x* + 10.25, with *r* = 0.987, respectively.

### 3.5. Statistical Analysis

The statistical analysis was carried out using SPSS 17.0 (SPSS Inc., Chicago, IL, USA, 2009) software. All data provided in this study was considered the mean and standard deviation of at least triplicate results. The noticeable variations in between the means were determined by Duncan’s Multiple Range Test.

## 4. Conclusions

Current study anticipated to elucidate the molecular characterization of the carotenoid biosynthetic pathway genes. In this study IdPSY, IdPDS, IdZDS, IdLCYB, IdCHXB, IdCHXE, and IdZEP, were cloned and characterized from *I. dentata* using NGS data. We have revealed the signature motifs of carotenoid biosynthic genes of *I. dentata* by in silico analysis. The carotenoid pathway genes transcript regulation and carotenoid accumulation in different organs of *I. dentata* was analyzed by qRT-PCR and HPLC, respectively. The maximum carotenoid content was found in the leaf which is consumed as a seasoned vegetable that might be useful to combat vitamin A deficiency. The analyzed information might be useful to resolve the carotenoid biosynthetic molecular mechanism in plants and especially in *I. dentata*. In addition to the medicinal properties of *I. dentata*, this data may contribute to elucidate the molecular insights of the carotenoid biosynthesis in different organs in plants.

## Figures and Tables

**Table 1:** Accumulation of carotenoids in different organs of *I. dentata*. Each value is the mean of three independent replications.

Name of the Carotenoid	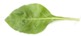	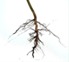	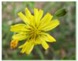
(**1**) 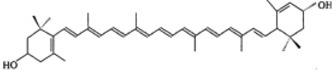	415.32 ± 32.62a	N/D	197.28 ± 36.22b
(**2**) 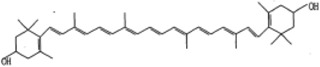	21.59 ± 0.94a	N/D	24.03 ± 3.41a
(**3**) 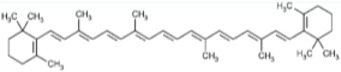	140.88 ± 16.79a	N/D	21.6 ± 3.95b
(**4**) 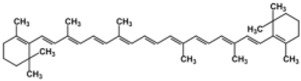	803.43 ± 61.5a	N/D	97.26 ± 16.91b
(**5**) 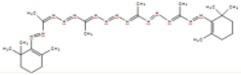	117.03 ± 4.2a	N/D	13.41 ± 2.33b
Total carotenoid	1498.25	N/D	353.59

Accumulation of carotenoid compounds in flower, leaf, and root of *I. dentata*. (μg/g DW); (**1**) Lutein; (**2**) Zeaxanthin; (**3**) 13Z-β-carotene; (**4**) β-carotene; (**5**) 9*Z*-β-carotene. Each value is the mean of three replications ± standard deviation; N/D not detected.

**Table 2:** Primers used in this study.

Primer Name	Sequence (5′ → 3′)	Size (bp)	GenBank Access. No
*IdPSY (F)*	CCGCAAGATGAGCTGGCGAA	179	MF611777
*IdPSY (R)*	GATGCCCAAACCGGCCATCT		
*IdPDS (F)*	GGAAAGGTGGCGGCTTGGA	136	MF611774
*IdPDS (R)*	GCTCCTTCCATTGCAATCGGTCAT		
*IdZDS (F)*	TGCTGTGGCTCTTGCTTTAAGTCC	160	MF611772
*IdZDS (R)*	TGCAACCGGGTCCCACATTC		
*IdLCYB (F)*	GGTCGCGGAGGTTGTGTCAA	174	MF611775
*IdLCYB (R)*	GACTTGTTGGGCGACTGCGA		
*IdCHXB (F)*	TGGCAAATGGAGGGTGGAGAGG	103	MF614116
*IdCHXB (R)*	GCGCCCATCTCGCCCAATAC		
*IdCHXE (F)*	TTCACTGTGGACGGCAAGGC	142	MF611776
*IdCHXE (R)*	AGCGGTGTCATTGCGTGCAT		
*IdZEP (F)*	GGAGGTGGGATCGGAGGGTT	155	MF611773
*IdZEP (R)*	GCTTCCAAAGCAGCCAACGC		
*IdActin (F)*	CAATGGAACCGGAATGGTCA	100	KY419710
*IdActin (R)*	CATTACGCCCGTGTGTCGAG		

## Supplementary Materials

Supplementary materials are available online.
