# Supplementary material for: Molecular Cloning and Characterization of Carotenoid Pathway Genes and Carotenoid Content in Ixeris dentata var. albiflora
Source: Molecules. 2017 Aug 31;22(9):1449. doi: 10.3390/molecules22091449 (PMC6151524; doi:10.3390/molecules22091449)
Supplement: Supplementary file 1 [file molecules-22-01449-s001.pdf]

Article

Molecular Cloning and Characterization of Carotenoid Pathway Genes and Carotenoid Content in *Ixeris dentata* var. *albiflora*

Chinreddy Subramanyam Reddy [1†], Sang-Hoon Lee [1†], Jeong Soo Yun [2], Jae Kwang Kim [2], Sang Won Lee [1], Mok Hur [1], Seong Cheol Koo [1], Mi Ran Kim [1], Woo Moon Lee [1], Jae Ki Jang [1], Yoonkang Hur [4], Yeon Bok Kim [1,3*]

1. Department of Herbal Crop Resources, National Institute of Horticultural & Herbal Science, RDA, Eumseong-gun, 27709, Korea; suumani@gmail.com; omega119@naver.com; swlee1004@korea.kr; mok0822@korea.kr; ksch992@korea.kr; milan80623@korea.kr; wmlee65@korea.kr; changjk@korea.kr; yeondarabok@korea.kr
2. Division of Life Sciences, College of Life Sciences and Bioengineering, Incheon National University, Incheon 22012, Korea; 210621036@inu.ac.kr; kjkpj@incheon.ac.kr
3. Department of Medicinal and Industrial Crops, Korea National College of Agriculture and Fisheries, Jeonju 54874, Korea; yeondarabok@korea.kr
4. Department of Biology, Chungnam National University, 99 Daehak-ro, Yuseong-gu, Daejeon 34134, Korea; ykhur@cnu.ac.kr

† This author is contributed equally to this work.

* Correspondence: E-Mail: yeondarabok@korea.kr; Tel.: +82-63-238-9092; Fax: +82-63-238-9080

Supplementary Figure Legends

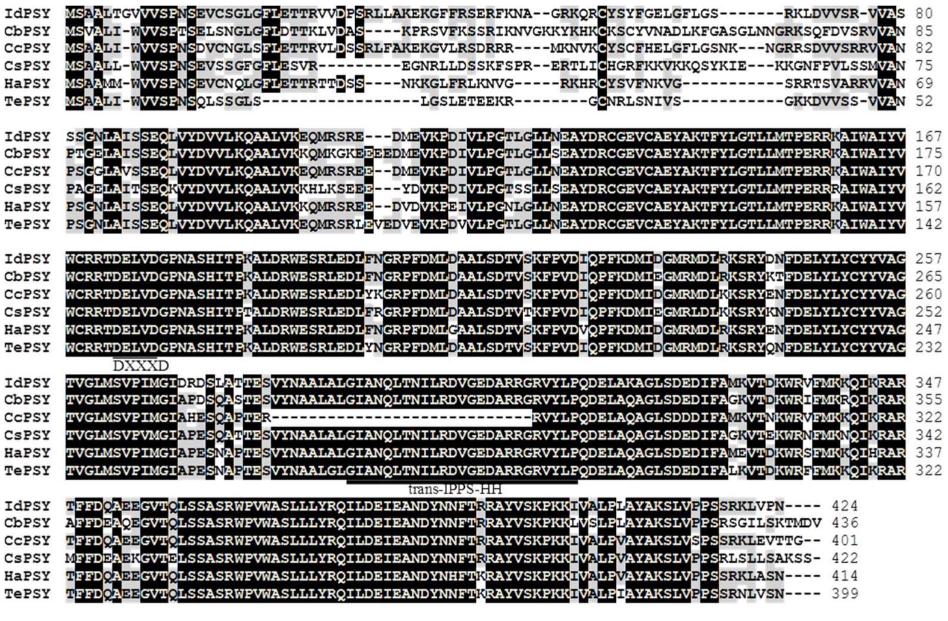

Supplementary Figure S1: Deduced amino acid multiple sequence alignments of the IdPSY with other PSYs. Identical amino acids are indicated by a black background, and similar residues are shaded with a gray background. The aspartate rich domain (DXXXD, where X represents any Amino Acid) was underlined and bold underline indicates trans-isoprenyl diphosphate synthase (trans-IPPS-HH) motif. CbPSY: *Chrysanthemum boreale* (AGU91437.1); CcPSY: *Cynara cardunculus var. scolymus*(KVI03037.1); CsPSY: *Camellia sinensis* (AJB84620.1); HaPSY: *Helianthus annuus* (CAC19567.1); TePSY: *Tagetes erecta* (AAM45379.1): IdPSY: *I. dentata* (MF611777).

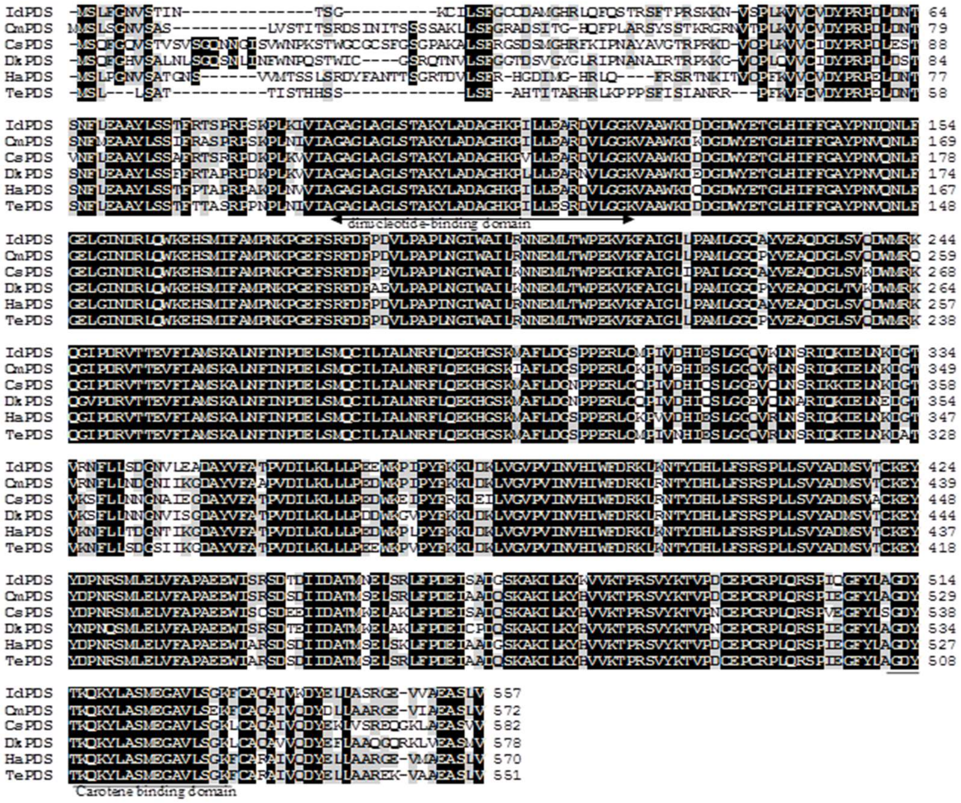

Supplementary Figure S2: Deduced amino acid multiple sequence alignments of the IdPDS with other PDSs. Identical amino acids are indicated by a black background and similar residues are shaded with a gray background. The arrowed black underline indicates the dinucleotide-binding domain and another underline represents carotene binding domain. CmPDS: *Chrysanthemum x morifolium* (BAE79552.1); CsPDS*: Camellia sinensis* (AHB32104.1); DkPDS: *Diospyros kaki* (ACY78343.1); HaPDS: *Helianthus annuus* (AHA36971.1); TePDS*: Tagetes erecta* (AAG10426.1); IdPDS: *I. dentata* (MF611774).

.

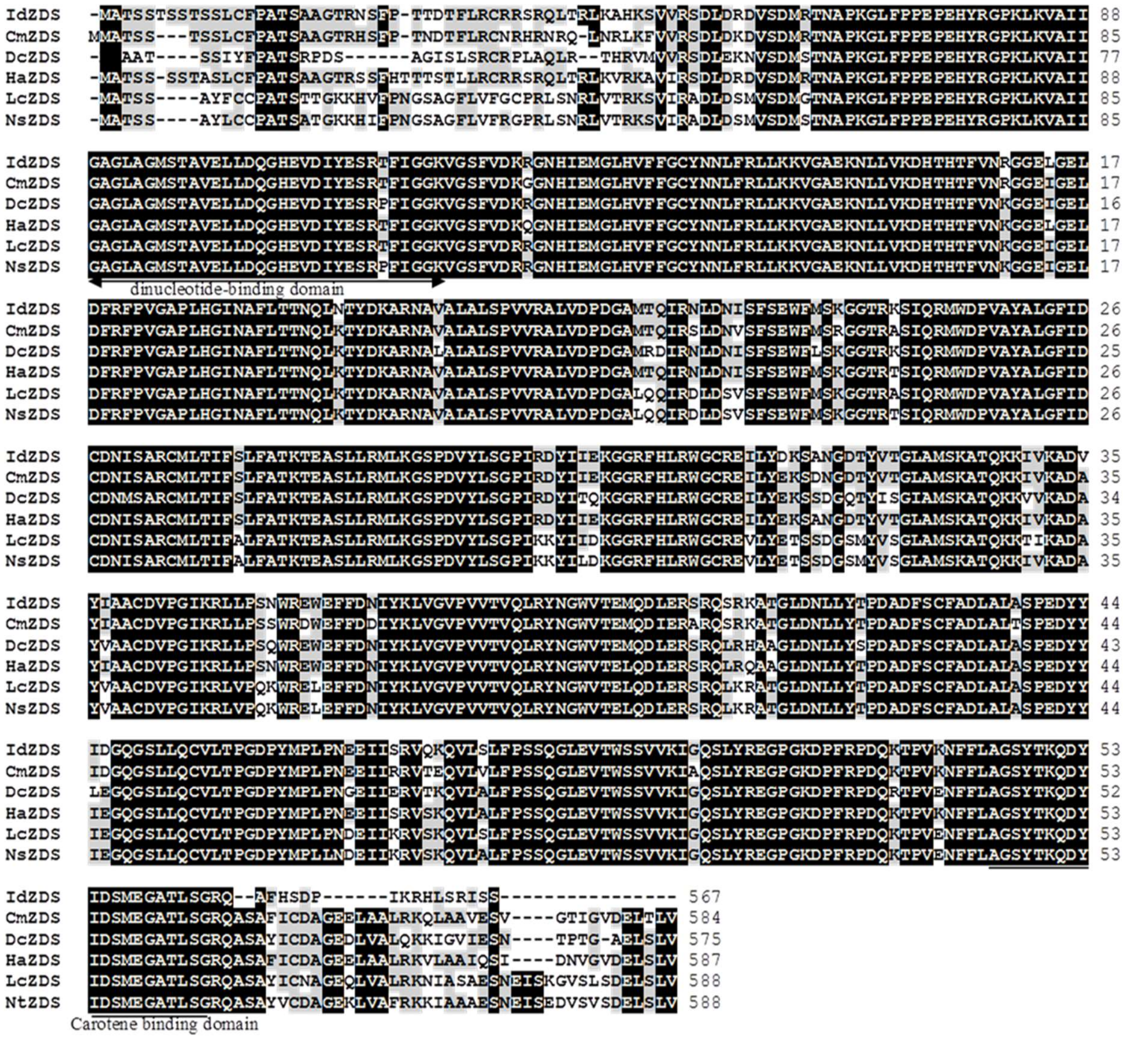

Supplementary Figure S3: Deduced amino acid sequence multiple alignments of the IdZDS with other ZDSs. Identical amino acids are indicated by a black background, and similar residues are shaded with a gray background. The arrowed black underline indicates the dinucleotide-binding domain and another underline represents carotene binding domain. CmZDS: *Chrysanthemum x morifolium* (BAE79555.1); DcZDS: *Daucus carota subsp. sativus (*NP_001316091.1); HaZDS: *Helianthus annuus (*AHA36972.1); LcZDS: *Lycium chinense (*AIZ50712.1); NtZDS: *Nicotiana tabacum (*AEG73891.1); IdZDS: *I. dentata* (MF611772).

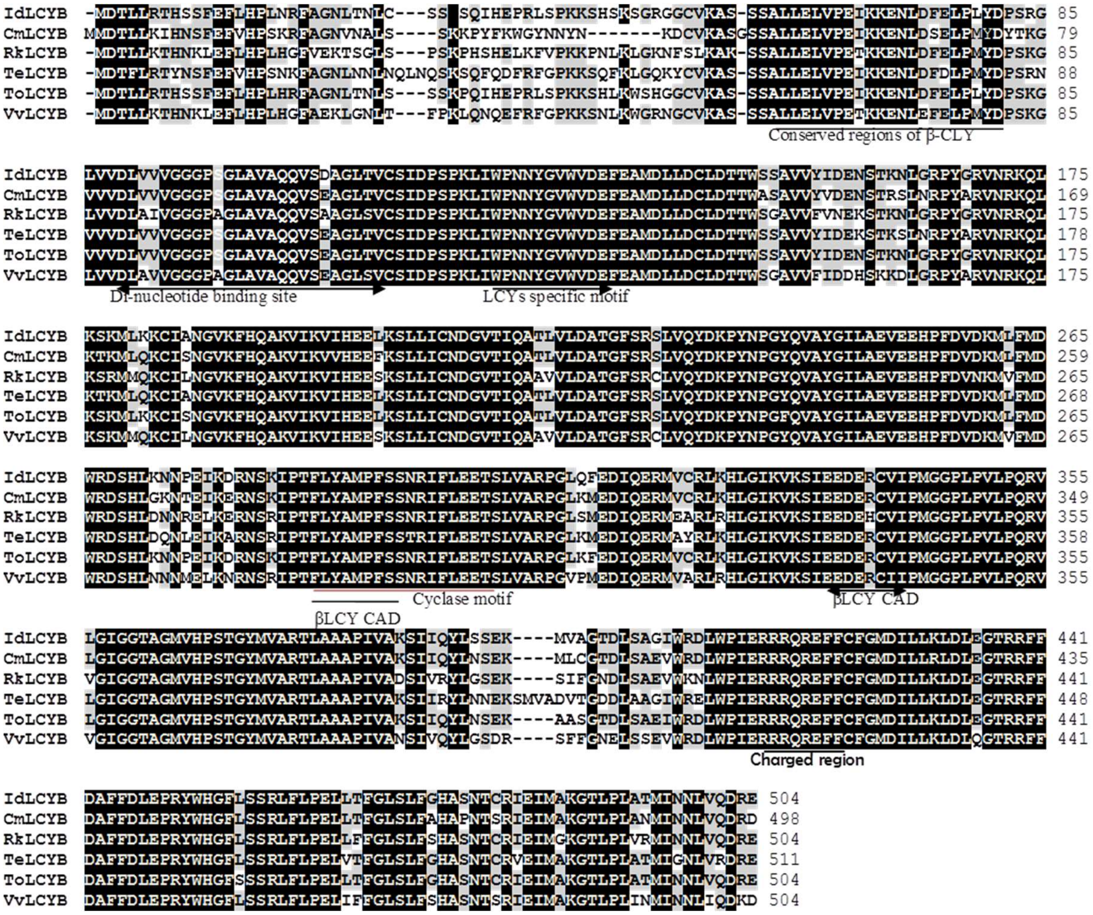

Supplementary Figure S4: Deduced amino acid multiple sequence alignment of IdLCYB with various LCYbs. Identical amino acids are indicated by a black background, and similar residues are shaded with a gray background. Conserved LCYB region, Di-nucleotide binding site, LCY's specific motif, Cyclase motif βLCY catalytic active domains and charged regions were underlined. CmLCYB: *Chrysanthemum x morifolium* (BAE79544.1); RkLCYB: *Rhododendron kiusianum x Rhododendron indicum* (BAS69436.1); TeLCYB: *Tagetes erecta* (ARR74226.1); ToLCYB: *Taraxacum officinale (*BAE78471.1); VvLCYB: *Vitis vinefera (*AFP28799.1); IdLCYB: *I. dentata* (MF611775).

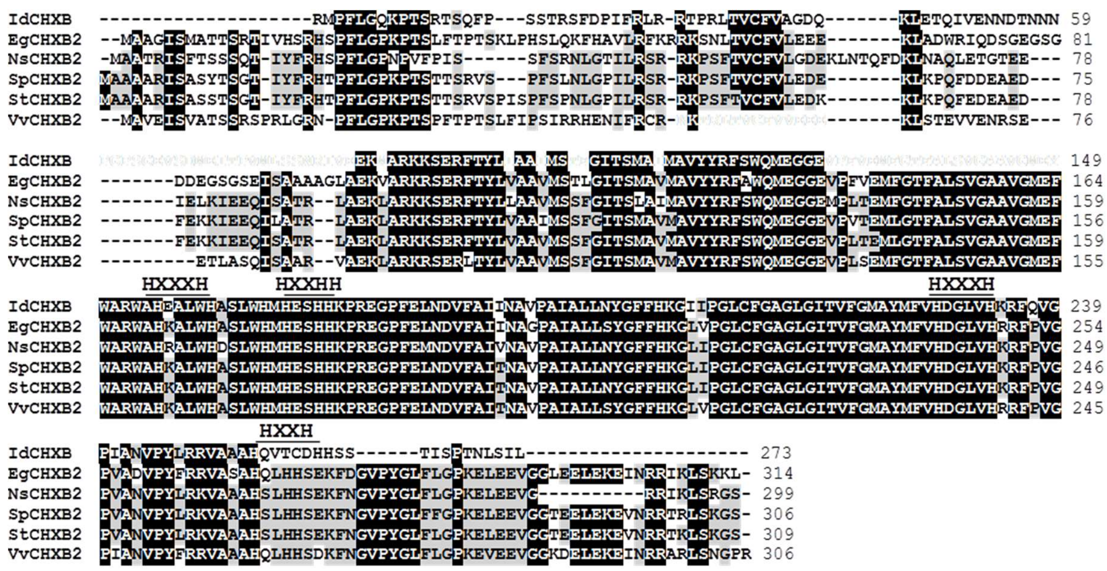

Supplementary Figure S5: Deduced amino acid multiple sequence alignment of IdCHXB with various CHXBs. Identical amino acids are indicated by a black background, and similar residues are shaded with a gray background. Spatially conserved four histidine domains believed to be participated in iron ion adhesion while hydroxylation are (HXXXH) and HXXH) where x indicated for any amino acid) marked with text. EgCHXB: *Erythranthe guttata (*XP_012858684.1); NsCHXB: *Nicotiana sylvestris* (XP_009780858.1); SpCHXB: *Solanum pennellii (*XP_015080043.1); StCHXB: *Solanum tuberosum* (XP_006360197.1); VvCHXB: *Vitis vinifera* (XP_002273581.1); IdCHXB: *I. dentata* (MF614116).

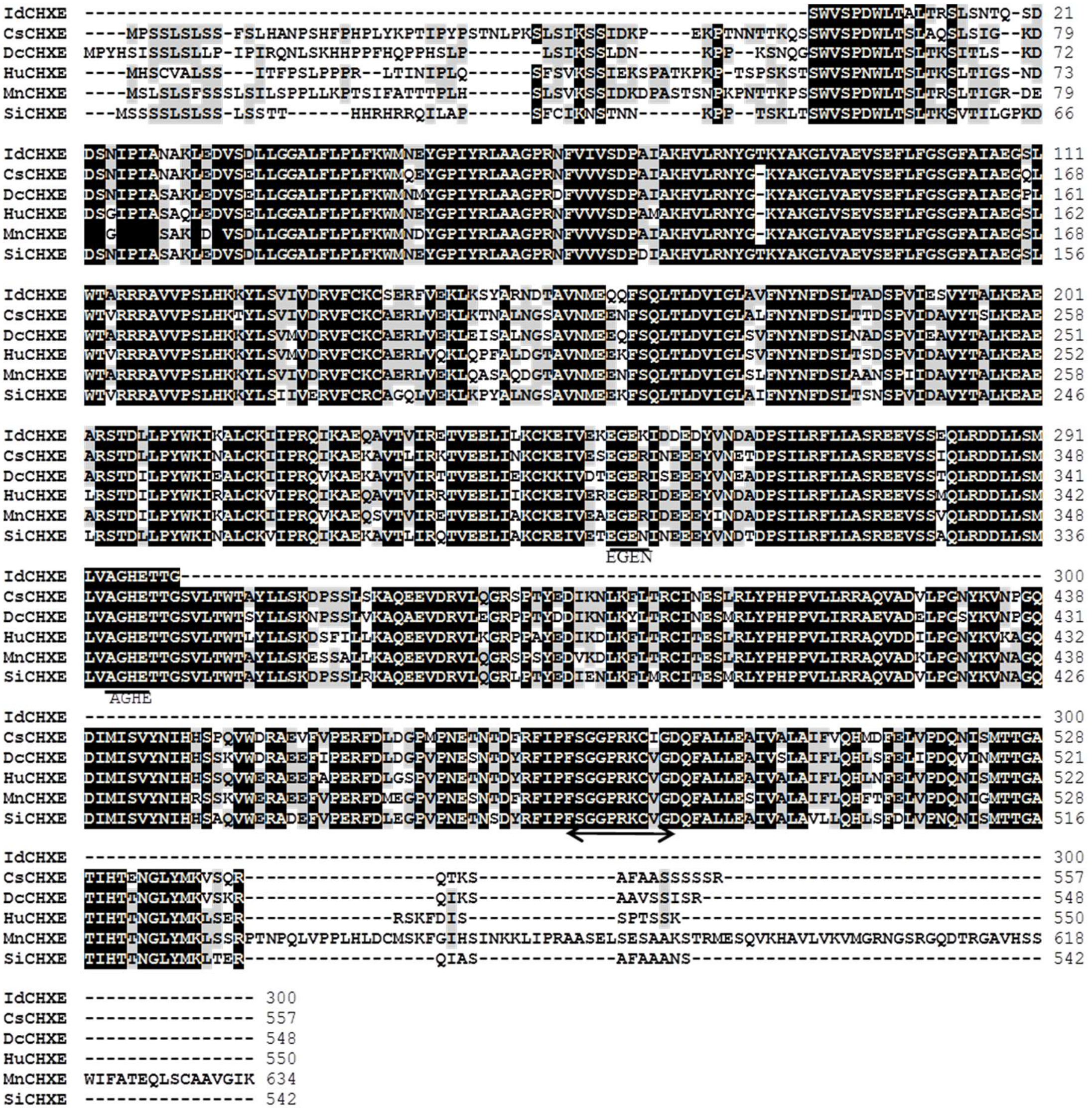

Supplementary Figure S6: Deduced amino acid multiple sequence alignments of the IdCHXE with other CHXE. Identical amino acids are indicated by a black background, and similar residues are shaded with a gray background. The sequence (AGHE) indicates center of α helix which can bind to heme and the sequence (EGEN) indicates salt bridges were underlined. The arrowed underline indicates heme binding region. CsCHXE: *Camellia sinensis* (AJB84623.1); DcCHXB: *Daucus carota subsp. sativus* (NP_001316100.1); HuCHXE: *Herrania umbratica* (XP_021289420.1); MnCHXE: *Morus notabilis* (XP_010098846.1); SiCHXE: *Sesamum indicum* (XP_011094564.1); IdCHXE: *I. dentata* (MF611776).

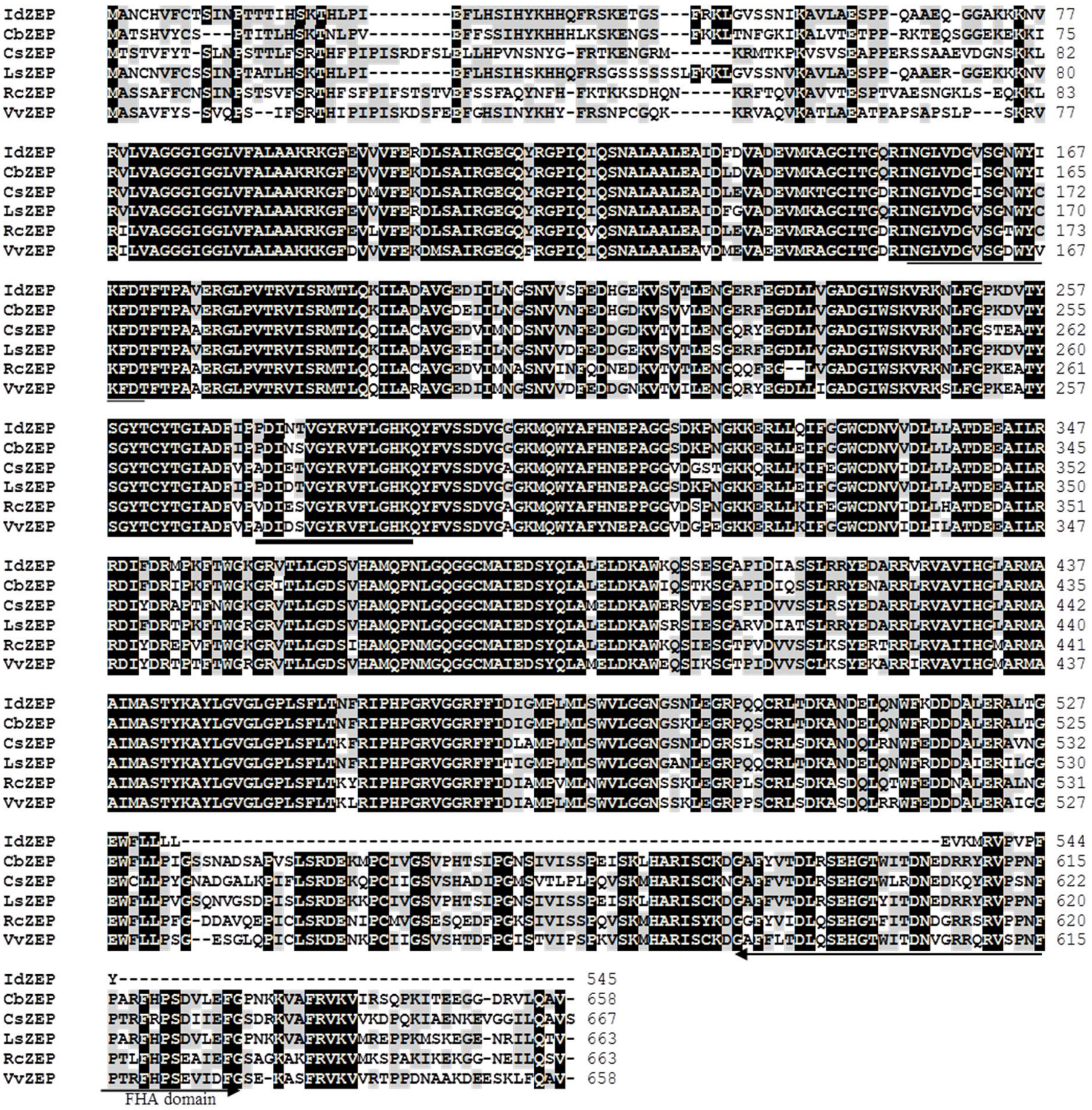

Supplementary Figure S7: Deduced amino acid sequences multiple alignments of the IdZEP with other ZEPs. Identical amino acids are indicated by a black background, and similar residues are shaded with a gray background. The solid black colour underline indicates the short motifs of lipocalin family proteins and Arrowed red underline represents FHA domain. CbZEP: *Chrysanthemum boreale* (AGU91434.1); CsZEP: *Camellia sinensis* (AJB84624.1); LsZEP*: Lactuca Sativa* (BAE72089.1); RcZEP: *Ricinus communis* (XP_002523587.1); VvZEP: *Vitis vinifera* (NP_001268202.1); IdZEP: *I. dentata* (MF611773).
